# Lipid trait-associated genetic variation is associated with gallstone disease in the diverse Third National Health and Nutrition Examination Survey (NHANES III)

**DOI:** 10.1186/1471-2350-14-120

**Published:** 2013-11-21

**Authors:** Robert Goodloe, Kristin Brown-Gentry, Niloufar B Gillani, Hailing Jin, Ping Mayo, Melissa Allen, Bob McClellan, Jonathan Boston, Cara Sutcliffe, Nathalie Schnetz-Boutaud, Holli H Dilks, Dana C Crawford

**Affiliations:** 1 Center for Human Genetics Research, Vanderbilt University, 2215 Garland Avenue, 519 Light Hall, Nashville, Tennessee 37232, USA; 2 Department of Molecular Physiology and Biophysics, Vanderbilt University, Nashville, Tennessee, USA

**Keywords:** Gallstones, NHANES III, PAGE, EAGLE, *ABCG5*, Genetic association

## Abstract

**Background:**

Gallstone disease is one of the most common digestive disorders, affecting more than 30 million Americans. Previous twin studies suggest a heritability of 25% for gallstone formation. To date, one genome-wide association study (GWAS) has been performed in a population of European-descent. Several candidate gene studies have been performed in various populations, but most have been inconclusive. Given that gallstones consist of up to 80% cholesterol, we hypothesized that common genetic variants associated with high-density lipoprotein cholesterol (HDL-C), low-density lipoprotein cholesterol (LDL-C), and triglycerides (TG) would also be associated with gallstone risk.

**Methods:**

To test this hypothesis, the Epidemiologic Architecture for Genes Linked to Environment (EAGLE) study as part of the Population Architecture using Genomics and Epidemiology (PAGE) study performed tests of association between 49 GWAS-identified lipid trait SNPs and gallstone disease in non-Hispanic whites (446 cases and 1,962 controls), non-Hispanic blacks (179 cases and 1,540 controls), and Mexican Americans (227 cases and 1,478 controls) ascertained for the population-based Third National Health and Nutrition Examination Survey (NHANES III).

**Results:**

At a liberal significance threshold of 0.05, five, four, and four SNP(s) were associated with disease risk in non-Hispanic whites, non-Hispanic blacks, and Mexican Americans, respectively. No one SNP was associated with gallstone disease risk in all three racial/ethnic groups. The most significant association was observed for *ABCG5* rs6756629 in non-Hispanic whites [odds ratio (OR) = 1.89; 95% confidence interval (CI) = 1.44-2.49; p = 0.0001). *ABCG5* rs6756629 is in strong linkage disequilibrium with rs11887534 (D19H), a variant previously associated with gallstone disease risk in populations of European-descent.

**Conclusions:**

We replicated a previously associated variant for gallstone disease risk in non-Hispanic whites. Further discovery and fine-mapping efforts in diverse populations are needed to fully describe the genetic architecture of gallstone disease risk in humans.

## Background

Gallstone disease has become one of the most common digestive disorders in the world. Gallstone disease affects more than 30 million Americans and accounts for 750,000 gallbladder removals in the United States annually and at least 190,000 gallbladder removals in European countries such as Germany [1]. The prevalence of gallstone disease varies by race/ethnicity with Native Americans and Hispanics having the highest reported prevalence compared with populations of European or African descent [2-7]. The incidence and prevalence is likely under-estimated given the fact that up to 80% of individuals with gallstones are asymptomatic or have non-traditional symptoms of disease [8].

There are a number of well-established gallstone disease risk factors including older age, female sex, increased body mass index, race/ethnicity, and certain dietary habits such as consumption of food rich in refined carbohydrates and lipids [1,9-13]. In addition to the established demographic and lifestyle risk factors, there is evidence that gallstone disease risk has a significant genetic component. Family history of gallstone disease increases an individual’s risk for the same disease [8,14,15]. Twin studies suggest that up to 25% of the risk of gallstone disease is due to genetic factors [16]. Indeed, recent candidate gene [17-19] and genome-wide association (GWA) [20] studies along with linkage studies [21] have identified common genetic variants associated with gallstone disease risk.

The majority of gallstones are composed of condensed bile components containing up to 80% cholesterol (termed “cholesterol gallstones”) [13]. Based on the composition of gallstones and recent results from genetic association studies, we hypothesized that common genetic variants associated with lipid profiles would be associated with gallstone disease risk. To test this hypothesis, we conducted a genetic association study for gallstone disease and 49 genetic variants previously associated with high-density lipoprotein cholesterol (HDL-C), low density lipoprotein cholesterol (LDL-C), and triglycerides (TG) [22] in the diverse Third National Health and Nutrition Examination Survey (NHANES III) as part of the Epidemiologic Architecture for Genes Linked to Environment (EAGLE) study [23]. Overall, we observed thirteen associations between gallstone disease and lipid-associated variants among the three racial/ethnic groups at a liberal significance threshold of 0.05 with the most significant association observed for *ABCG5* rs6756629 in non-Hispanic whites (OR = 1.89; 95% CI: 1.44-2.49). *ABCG5* rs6756629 has been previously associated with lipid profiles (LDL-C, total cholesterol, and triglycerides) and is in strong linkage disequilibrium with *ABCG5* rs112887534 in European-descent populations [24], a variant recently associated with gallstone disease [20]. Collectively, our data support the hypothesis that genetic variation associated with lipid trait variation is also associated with gallstone disease risk.

## Methods

### Study population and phenotypes

The study population described here includes phase 2 participants ascertained between 1991 and 1994 as part of the Third National Health and Nutrition Examination Survey (NHANES III). NHANES is now conducted yearly by the National Center on Health Statistics (NCHS) at the Center for Disease Control and Prevention (CDC) with the major goal of assessing the health and nutrition status of Americans regardless of health status. Beginning with NHANES III phase 2, CDC began collecting bio-specimens from consenting participants for genetic studies. NHANES is a complex survey design that oversamples specific age groups (such as the elderly) and racial/ethnic groups (such as non-Hispanic blacks).

All eligible participants were given an approximately hour long interview. Participants also underwent a health examination by the Mobile Exam Centers (MEC), and blood and urine samples were also obtained [25]. Participants were eligible for NHANES at two months of age or older, while participants consenting for bio-specimen collection for DNA extraction had to be at least twelve years of age. Non-eligible individuals included military personnel and institutionalized civilians.

Genetic NHANES III includes 7,159 participants, of which 2,631 are self-described non-Hispanic white, 2,108 are self-described non-Hispanic black, and 2,073 are self-described Mexican American. All procedures were approved by the CDC Ethics Review Board and written informed consent was obtained from all participants. Because no identifying information was accessed by the investigators, Vanderbilt University’s Institutional Review Board determined that this study met the criteria of “non-human subjects.”

Gallstones were defined by a positive “yes” response to an administered questionnaire exam asking, “Has the doctor ever told you that you had gallstones?” or a positive ultrasound reading (presence of scar tissue (surgical removal of gallbladder or cholecystectomy) or demonstration of gallstones present in the gallbladder) from trained technicians. Controls were defined as gallstone-free participants who answered “no” response to the administered questionnaire or had no history of gallstone removal surgeries. To assess the potential for misclassification, we examined participants with both ultrasound and questionnaire data to validate asymptomatic cases and to identify misclassified controls.

### SNP selection and genotyping

A total of 49 GWAS-identified SNPs were selected for study based on evidence of being previously associated with at least one lipid profile (HDL-C, LDL-C, TG) in published candidate gene and genome-wide association studies [10,12,18,20,22]. The SNP selection criteria meet the genome-wide significance of p < 10^-8^ in previous published studies [22]. SNP data was generated by genotyping using Sequenom and Illumina BeadXpress platforms. Thorough genotyping and SNP details (gene region, physical location, coding type, etc.) have been previously published in Text S1 of [22]. CDC quality control metrics were implemented on the 49 SNPs and included tests of Hardy-Weinberg Equilibrium (threshold of p > 0.0001 in at least two of the three race/ethnicities) as well as concordance with blinded duplicates supplied by CDC (at least 95%). Also, as part of the PAGE study [23], we genotyped these lipid-associated SNPs in 360 HapMap samples for network-wide quality control assessment.

### Statistical methods

Participants less than 18 years of age were excluded from this study. Single SNP tests of association were performed using logistic regression. Gallstone status (yes/no) was the dependent variable and each SNP assuming an additive genetic model was the independent variable. All models were adjusted for age, sex, and body mass index. Logistic regressions were performed stratified by self-reported race/ethnicity, and genetic effect estimates were expressed as odds ratios (ORs) with 95% confidence intervals (95% CIs). All analyses were conducted in SAS v9.2 (SAS Institute, Cary, NC) using the Analytic Data Research by Email (ANDRE) portal of the CDC Research Data Center in Hyattsville, MD. Results were plotted using Synthesis-View [26]. Linkage disequilibrium was assessed using SNP Annotation and Proxy Search (SNAP) [27].

## Results

### Study population characteristics

Study population characteristics by self-described race/ethnicity and gallstone disease status are given in Table 1. Overall, we identified 446, 179, and 227 cases of gallstone disease among non-Hispanic whites, non-Hispanic blacks, and Mexican Americans, respectively. As expected, based on the known epidemiology of gallstone disease [3,13], cases tended to be female, older, and have a higher BMI compared with controls (Table 1). Also, overall, there were proportionally fewer cases among non-Hispanic blacks compared with non-Hispanic whites and Mexican Americans, which is consistent with the lower prevalence of this disease among this group [3].

**Table 1:** Study population characteristics by gallstone disease status stratified by race/ethnicity

	**Non-hispanic whites**			**Non-hispanic blacks**			**Mexican Americans**		
	**n = 2,408**			**n = 1,719**			**n = 1,708**		
	**Cases**	**Controls**	**P-value**	**Cases**	**Controls**	**P-value**	**Cases**	**Controls**	**P-value**
**n**	446	1,962	-	179	1,540	-	227	1,478	-
**% Female**	71.13%	58.10%	5.24E-07	76.54%	56.17%	3.66E-07	73.29%	45.81%	5.55E-16
**Age in years**	61.74	51.91	6.49E-23	49.67	40.02	1.11E-16	53.80	39.27	2.59E-33
	(16.45)	(20.61)		(16.71)	(16.48)		(15.16)	(16.95)	
**BMI**	28.51	26.24	5.13E-14	30.77	27.96	2..32E-07	30.27	27.22	3.33E-16
**(kg/m**^**2**^**)**	(6.1)	(5.35)		6.67)	(6.61)		(5.86)	(5.2)	

Abbreviations: *BMI* body mass index.

Means (and standard deviations) are given unless otherwise noted. P-values were calculated based on the Wald test using unweighted survey data.

Among the 49 lipid trait-associated SNPs tested for association with gallstone disease, five, four, and four SNPs were associated with disease risk at a liberal significance threshold of 0.05 among non-Hispanic whites, non-Hispanic blacks, and Mexican Americans, respectively (Table 2; Additional file 1: Table S1). Among all groups, the most significant finding was observed in non-Hispanic whites (OR = 1.89; 95% CI = 1.44-2.49; p = 0.0001) for non-synonymous *ABCG5* rs6756629, a variant whose minor allele (A) was previously associated with decreased LDL-C and total cholesterol and increased triglycerides among European-descent populations [28]. This associated SNP is in strong linkage disequilibrium with *ABCG5* rs11887534 identified in a genome-wide association within a European-descent population for gallstone disease [20,24]. The second association identified for gallstone disease in non-Hispanic whites involved *ABCG8* rs6544713 (OR = 1.35; 95% CI = 1.14-1.61; p = 0.0007), a variant previously associated with LDL-C levels in European-descent populations [29]. *ABCG8* rs6544713 is not in linkage disequilibrium with rs6756629 or rs11887534 (both r^2^ = 0.047 in CEU 1000 Genomes Project) and therefore most likely represents an independent association with gallstone disease risk.

**Table 2:** Significant tests of association for gallstone disease by population

**SNPs**	**Gene**	**OAT**	**Non-Hispanic Whites**			**Non-Hispanic Blacks**			**Mexican Americans**		
			**Case/ Control CAF**	**OR (95% C.I.)**	**P**	**Case/ Control CAF**	**OR (95% C.I.)**	**P**	**Case/ Control CAF**	**OR (95% C.I.)**	**P**
rs10401969	*SUGP1*	TG	§	1.37 (1.05, 1.78)	***1.89E-02***	§	1.01 (0.74, 1.38)	9.61E-01	§	0.69 (0.41, 1.15)	1.52E-01
rs10889353	*DOCK7*	TG	0.64/ 0.67	0.90 (0.76, 1.05)	1.83E-01	0.61/ 0.62	0.84 (0.67, 1.06)	1.50E-01	0.66/ 0.62	1.32 (1.05, 1.67)	***1.73E-02***
rs17216525	*CILP2*	TG	§	0.76 (0.58, 0.99)	***4.45E-02***	§	1.26 (0.64, 2.46)	5.02E-01	§	0.88 (0.53, 1.45)	6.15E-01
rs1883025	*ABCA1*	HDL	0.27/ 0.26	1.09 (0.92, 1.30)	3.36E-01	0.30/ 0.33	0.84 (0.66, 1.09)	1.86E-01	0.26/ 0.28	0.78 (0.62, 0.98)	***3.30E-02***
rs2650000	*HNF1A LEF1*	LDL	0.66/ 0.65	1.146 (0.97, 1.35)	1.02E-01	§	1.50 (1.02, 2.21)	***3.73E-02***	0.63/ 0.63	0.976 (0.79, 1.20)	8.21E-01
rs28927680	*BUD13*	HDL TG	0.09/ 0.07	1.23 (0.93, 1.62)	1.53E-01	0.22/ 0.17	1.40 (1.06, 1.86)	***1.92E-02***	0.16/ 0.14	1.07 (0.81, 1.42)	6.27E-01
rs3764261	*CETP*	HDL	0.68/ 0.68	0.95 (0.80, 1.12)	5.15E-01	0.63/ 0.69	0.71 (0.56, 0.90)	***4.70E-03***	0.66/ 0.66	1.06 (0.85, 1.33)	5.89E-01
rs4149268	*ABCA1*	HDL	0.38/ 0.38	1.10 (0.94, 1.30)	2.34E-01	0.69/ 0.65	1.03 (0.81, 1.31)	8.21E-01	0.30/ 0.33	0.75 (0.60, 0.95)	***1.53E-02***
rs4939883	*LIPG*	HDL	0.83/ 0.83	1.28 (1.03, 1.59)	***2.71E-02***	0.56/ 0.54	0.94 (0.75, 1.19)	6.17E-01	§	0.88 (0.65, 1.19)	4.14E-01
rs6544713	*ABCG8*	LDL	0.71/ 0.68	1.35 (1.14, 1.61)	***7.00E-04***	§	1.19 (0.88, 1.62)	2.65E-01	0.85/ 0.82	1.22 (0.92, 1.60)	1.66E-01
rs6756629	*ABCG5*	LDL	0.09/ 0.06	1.89 (1.44, 2.49)	***1.00E-04***	0.13/ 0.08	1.24 (0.82, 1.87)	3.03E-01	0.12/ 0.09	1.19 (0.85, 1.67)	3.04E-01
rs754523	*APOB*	LDL	0.33/ 0.32	1.02 (0.86, 1.20)	8.34E-01	0.27/ 0.22	1.36 (1.04, 1.78)	***2.41E-02***	0.24/ 0.29	0.86 (0.68, 1.08)	1.82E-01
rs7679	*PCIF1*	HDL	0.19/ 0.16	0.97 (0.79, 1.19)	7.45E-01	§	1.18 (0.75, 1.84)	4.73E-01	0.14/ 0.11	1.51 (1.13, 2.01)	***4.80E-03***

Single SNP tests of association were performed using logistic regression assuming an additive genetic model. Only results for SNPs associated at p < 0.05 in any one race/ethnicity are shown (bolded and italicized). Results displayed here were adjusted for age, sex, and body mass index. §Per data use agreement, coded allele frequencies are not outputted by CDC for cells with ≤5 counts.

Abbreviations: Original associated Trait *(OAT)*; High density lipoprotein cholesterol *(HDL-C)*; low density lipoprotein cholesterol *(LDL-C)*; triglycerides *(TG)*; coded allele frequency *(CAF)*; odds ratio *(OR)*; confidence interval *(CI)*; p-value *(P)*.

Interestingly, despite the smaller sample size, four associations at p < 0.05 were observed among non-Hispanic blacks. The most significant finding among non-Hispanic blacks was *BUD13* rs28927680 (OR = 1.40; 95% CI = 1.06-1.86, p = 0.019) previously associated with HDL-C and TG in European-descent populations [29]. Other nominal associations observed in non-Hispanic blacks included genetic variants previously associated with HDL-C (*CETP* rs3764261 (OR = 0.71; 95% CI 0.56-0.9)) and LDL-C (*HNF1A/LEF1* rs2650000 (OR = 1.50; 95% CI 1.02-2.21) and *APOB* rs754523 (OR = 1.36; 95% CI 1.04-1.78)).

Among Mexican Americans, four associations were nominally associated with gallstone disease risk: HDL-C (ABCA1 rs1883025 (OR = 0.78; 95% CI 0.62-0.98; p = 0.033), ABCA1 rs4149268 (OR = 0.75; 95% CI 0.60-0.95; p = 0.015), and *PCIF1* rs7679 (OR = 1.51; 95% CI 1.13-2.01; p = 0.048)) and TG (DOCK7 rs10889353 (OR = 1.23; 95% CI 1.05-1.67; p = 0.017). No association was associated at p < 0.05 across all three racial/ethnicity groups (Table 2).

**Figure 1:**
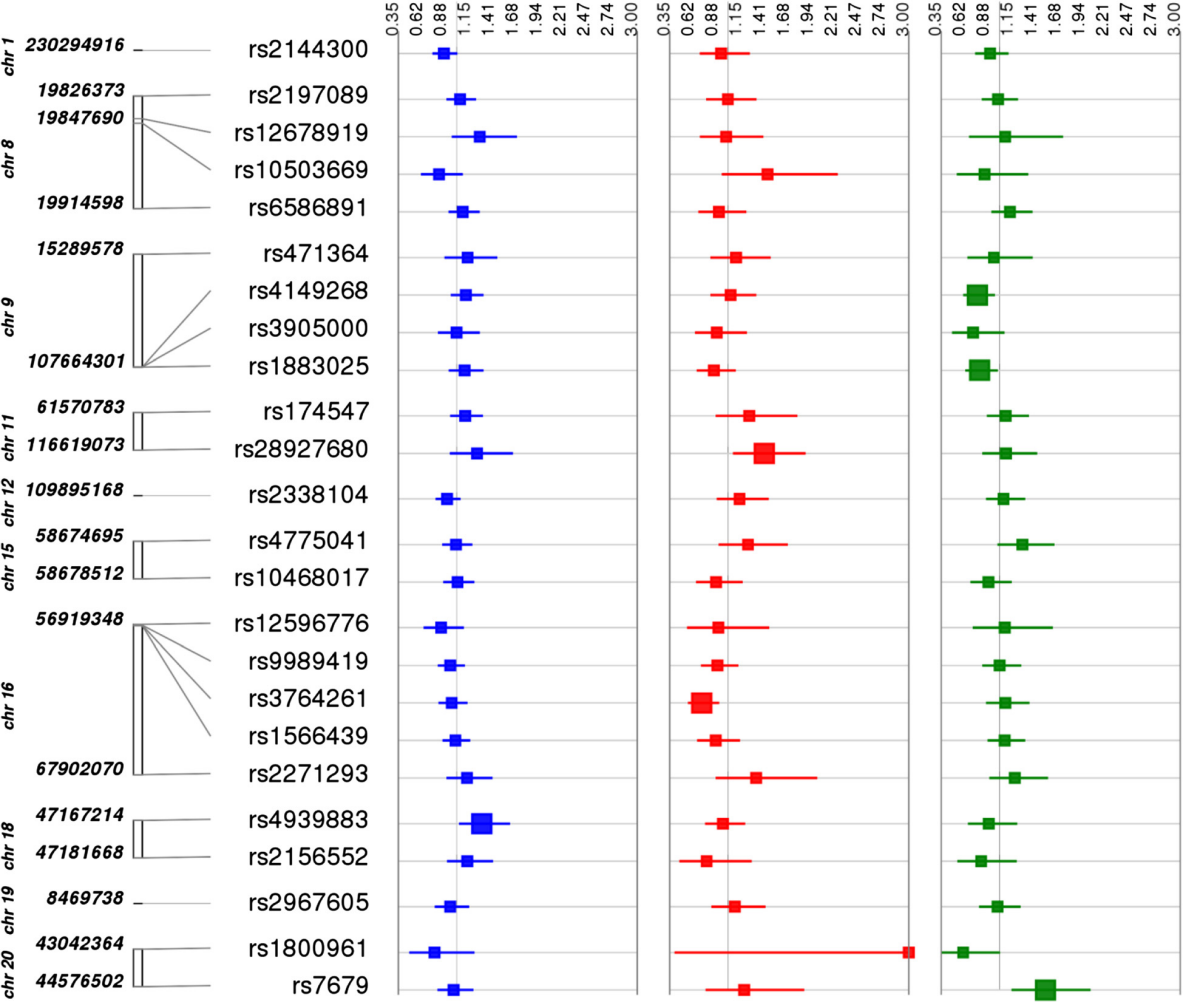
**Associations between HDL-C associated SNPs and gallstone disease by population.** Each high-density lipoprotein (HDL)-associated SNP was tested for an association with gallstone disease (yes/no) assuming an additive genetic model adjusted for age, sex, and body mass index [kg/m^2^]. The odds ratio and 95% confidence intervals are plotted by race-ethnicity using Synthesis-View [26]. SNP locations (genome build 37.5) are given on the y-axis. Each square represents an odds-ratio and each line represents a 95% confidence interval for each population. The larger square represents a significantly associated SNP at p < 0.05. Populations are color-coded as follows: non-Hispanic whites (blue), non-Hispanic blacks (red), and Mexican Americans (green). The grey vertical line represents the 1.0 threshold for odds-ratio values.

**Figure 2:**
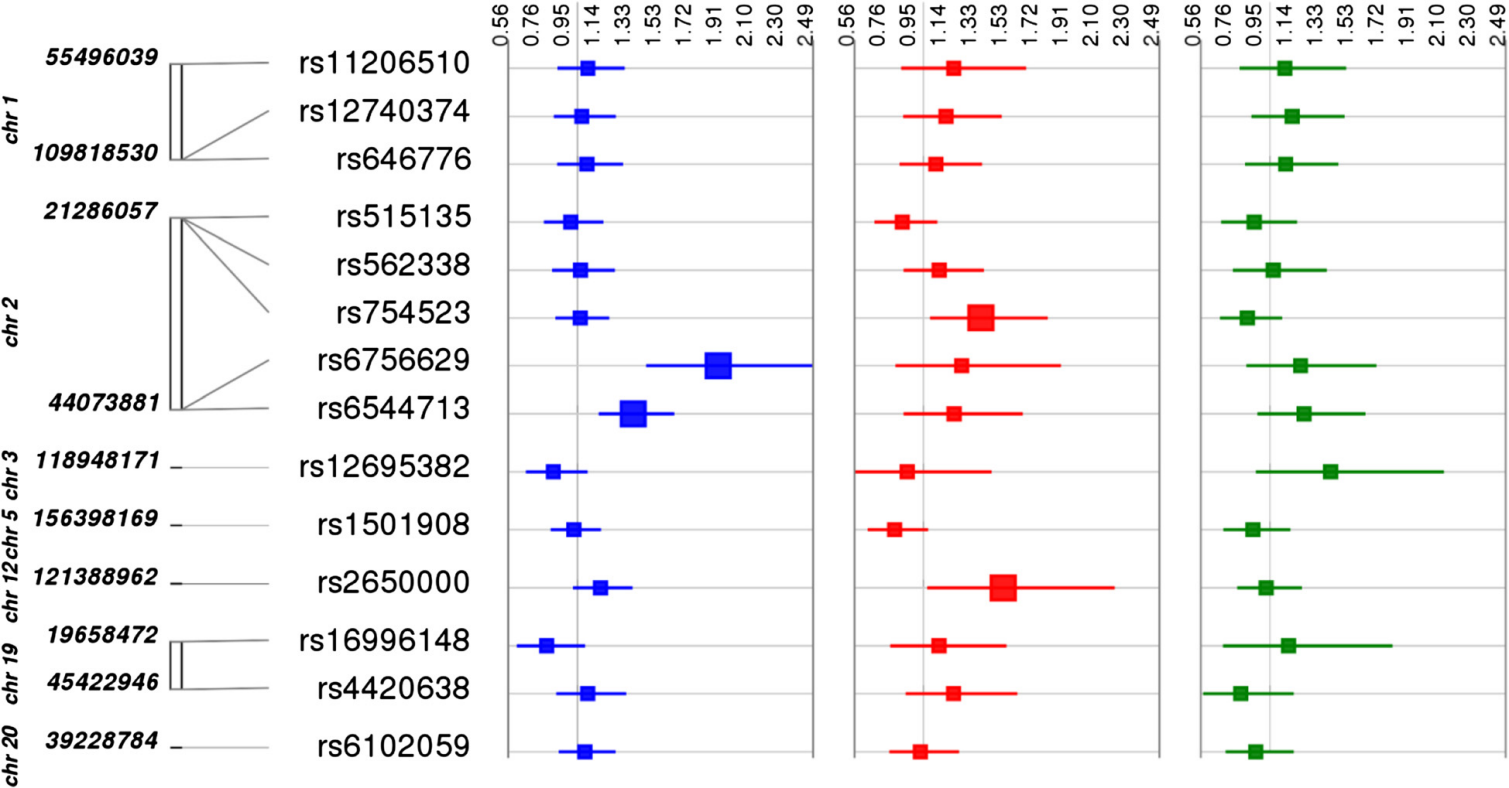
**Associations between LDL-C associated SNPs and gallstone disease by population.** Each low-density lipoprotein (LDL)-associated SNP was tested for an association with gallstone disease (yes/no) assuming an additive genetic model adjusted for age, sex, and body mass index [kg/m^2^]. The odds ratio and 95% confidence intervals are plotted by race-ethnicity using Synthesis-View [26]. SNP locations (genome build 37.5) are given on the y-axis. Each square represents an odds-ratio and each line represents a 95% confidence interval for each population. The larger square represents a significantly associated SNP at p < 0.05. Populations are color-coded as follows: non-Hispanic whites (blue), non-Hispanic blacks (red), and Mexican Americans (green). The grey vertical line represents the 1.0 threshold for odds-ratio values.

**Figure 3:**
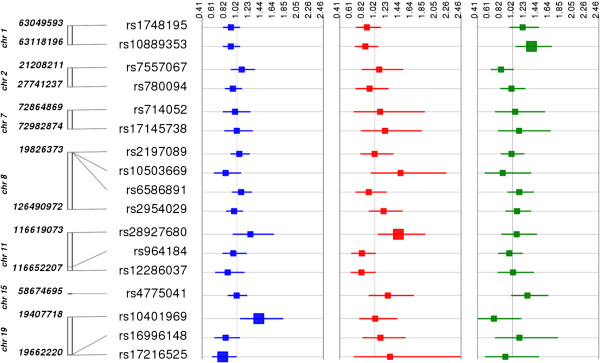
**Associations between TG associated SNPs and gallstone disease by population.** Each triglyceride (TG)-associated SNP was tested for an association with gallstone disease (yes/no) assuming an additive genetic model adjusted for age, sex, and body mass index [kg/m^2^]. The odds ratio and 95% confidence intervals are plotted by race-ethnicity using Synthesis-View [26]. SNP locations (genome build 37.5) are given on the y-axis. Each square represents an odds-ratio and each line represents a 95% confidence interval for each population. The larger square represents a significantly associated SNP at p < 0.05. Populations are color-coded as follows: non-Hispanic whites (blue), non-Hispanic blacks (red), and Mexican Americans (green). The grey vertical line represents the 1.0 threshold for odds-ratio values.

In addition to examining genetic associations for gallstone disease risk by race/ethnicity, we also examined the associations by associated lipid trait (Figures 1, 2 and 3; Additional file 2: Figure S1). That is, among the 49 SNPs tested here, 24, 14, and 17 SNPs were reported to be associated with HDL-C, LDL-C, and TG levels, respectively. As previously mentioned, the most significant finding involved an LDL-C associated variant, and, overall, approximately one-third (4 out of 14 or 29%) of LDL-C associated SNPs were nominally associated with gallstone disease risk in any one population. The other nominal findings reported at p < 0.05 were associated with HDL-C (6 out of 24 or 25%) or triglycerides (4 out of 17 or 24%) associated SNPs.

## Discussion

Among the 49 lipid-trait associated SNPs tested here, thirteen were associated with gallstone disease risk at a liberal significance threshold of 0.05 in at least one racial/ethnic group. Most (5/13; 38%) associations were observed among non-Hispanic whites; conversely, four associations each were identified among non-Hispanic blacks and Mexican Americans. None of the associations were observed in all three racial/ethnic groups. The strongest association for gallstone disease risk was observed in non-Hispanic whites (OR = 1.89; 95% CI: 1.44-2.49) for *ABCG5* rs6756629; although not significant (p = 0.30), this association trended in the same direction in non-Hispanic blacks (OR = 1.24; 95% CI 0.82-1.87) and Mexican Americans (OR = 1.26; 95% CI 0.89-1.79).

### Replication in European-descent populations

*ABCG5* rs6756629 (R50C) is in strong linkage disequilibrium with rs11887534 (D19H), a variant previously associated with gallstone disease risk in populations of European-descent [20]. Pairwise linkage disequilibrium (r^2^) was 1.00 in CEU from the 1000 Genomes Project Pilot Study [30] and 0.95 in a German study population [24]. Thus, the association observed here with rs6756629 likely represents a replication of the association identified in the original GWAS [20]. The magnitude of effect (OR = 1.89) is somewhat smaller compared with the original (OR = 2.2) [20] and subsequent reports [18,21,24,31], but the confidence intervals overlap suggesting similar overall genetic effect sizes for this population.

### Associations among non-European-descent populations

Very few genetic association studies and no GWA studies of gallstone disease risk have been performed in populations of non-European descent. A few studies have examined the association between *ABCG5* rs11887534 and gallstone disease in populations from China [24], Taiwan [32], and India [24]. To our knowledge, no study has examined *ABCG5* rs11887534 or tagged rs6756629 in African Americans or Mexican Americans for gallstone disease risk. As expected based on the known epidemiology of gallstone disease, the sample sizes available for non-Hispanic blacks (n = 179 cases) and Mexican Americans (n = 227 cases) were smaller compared with non-Hispanic whites (n = 446 cases). *ABCG5* rs6756629 was not associated with gallstone disease risk in non-Hispanic blacks (Table 2). The *ABCG5* variants rs11887534 and rs6756629 have lower pair-wise linkage disequilibrium in African-descent populations (YRI r^2^ = 0.613) compared with European-descent populations (CEU r^2^ = 1.000) based on data from the 1000 Genomes Project Pilot Study [30]. Recent statistical and functional data suggest that *ABCG5* rs11887534 (D19H) is likely the functional variant responsible for the association with gallstone disease risk while rs6756629 is likely a tagSNP [24]. The smaller sample sizes available for non-European descent populations coupled with differences in linkage disequilibrium between the genotyped rs6756629 and putative functional rs11887534 likely resulted in low statistical power to detect this association in non-Hispanic blacks and Mexican Americans. Further *ABCG5* fine-mapping and functional studies are needed to fully catalogue gallstone disease risk variants in diverse populations.

### Limitations and strengths

This study has several limitations and strengths. A major limitation is sample size and power. Although genetic NHANES III is large overall (n = 7,159), the number of cases and controls available for this gallstone disease study is small when stratified by race/ethnicity. More recent NHANES did not collect survey or ultrasound data; therefore, large sample sizes or independent replication datasets for genetic associations for gallstone disease risk are not available in NHANES. To maximize the power of the present study within NHANES III, we used both questionnaire and ultrasound data (where ultrasound data represented the “truth” or gold standard when both were available per participant) to define cases and controls. Indeed, limiting the dataset to participants with both data types would have resulted in a 14% reduction in sample size. A trade-off of this approach to maximize sample size, however, is that we may have misclassified participants who only had questionnaire data but no corroborating ultrasound data. To examine the possible extent of misclassification, we compared our case/control definition (questionnaire or ultrasound data) with case/control status among participants with both data types (excluding those participants with questionnaire data only). Overall, we found little evidence for misclassification using questionnaire data only (positive predictive value = 99%, negative predictive value = 100%, sensitivity = 100%, and specificity = 99%). We also compared results of tests of association between the two definitions and found little difference (Additional file 3: Figure S2).

Another limitation to the study is that we did not adjust our significance level for multiple statistical tests. Our liberal significance threshold coupled with the lack of an independent dataset can be cause for concerns for false positive associations. However, the most significant association reported here replicated an association previously reported in a GWAS in a population of similar race/ethnicity. The association between rs6756629 and gallstone disease risk in non-Hispanic whites, along with the association involving *ABCG5* rs6544713, would be considered significant with a conservative Bonferroni correction. The facts that the associations represent a replication of a previous report [20], that rs6756629 tags a likely functional SNP associated with gallstone disease risk [24], and that both survive correction for multiple testing support the conclusion that these are not a false positive findings.

A major strength of the current study is its diversity. Little to no data exist for the genetics of gallstone disease risk in African Americans and Mexican Americans. Further discovery studies are needed to identify the full genetic architecture of gallstone disease risk in diverse populations [33]. Another major strength of this current study is the combination of questionnaire and ultrasound data available in NHANES III. For participants with overlapping questionnaire and ultrasound data, we were able to identify asymptomatic cases of gallstones that would have otherwise been misclassified using questionnaire data only. Misclassification of case/control status lowers power to detect genetic associations; therefore, careful phenotyping is one of many essential components in the conduction of genetic association studies [34]. Combination of these data types maximized sample size for this study whereas restricting eligible cases and controls to participants with ultrasound data only would have reduced the sample size by 14% and therefore reduced power.

## Conclusions

In summary, we demonstrate here that lipid-trait associated genetic variants such as *ABCG5* rs6756629 are associated with gallstone disease risk. Larger studies of diverse populations are needed to determine the full spectrum of the genetic variants that contribute to gallstone disease risk in humans.

## Competing interests

The authors declare that they have no competing interests.

## Authors’ contributions

RG and DCC designed the study, interpreted the data, and drafted and edited the manuscript. RG and KB-G conducted the analyses. NBG, HJ, PM, MA, CS, NS-B, and HHD generated the genetic data and performed quality control analyses. BM and JB developed the analysis pipeline and maintained the study databases. All authors have read and approved the final manuscript.

## Pre-publication history

The pre-publication history for this paper can be accessed here:

http://www.biomedcentral.com/1471-2350/14/120/prepub

## Supplementary Material

Additional file 1: Table S1. All tests of association for gallstone disease by population. Single SNP tests of association were performed using logistic regression assuming an additive genetic model. Results displayed here were adjusted for age, sex, and body mass index. SNP position is based on genome build 37.5. Click here for file

Additional file 2: Figure S1 All associations between lipid trait-associated SNPs and gallstone disease by population. A total of 49 lipid-trait associated SNPs were tested for an association with gallstone disease using logistic regression adjusted for age, sex, and body mass index [kg/m^2^]. Synthesis-View [26] was used to display the results. SNP location (genome build 37.5) is given on the x-axis and p-values (-log_10_ transformed) are plotted along the y-axis at the top of the figure, while coded allele frequencies (CAF) are plotted along the y-axis at the bottom of the figure. Each triangle represents a p-value and each circle represents a CAF for each race-ethnicity. Populations are color-coded as follows: non-Hispanic whites (blue), non-Hispanic blacks (red), and Mexican Americans (green). The direction of the arrows corresponds to the direction of the beta coefficient. The significance threshold is indicated by the red bar at p = 0.05. Click here for file

Additional file 3: Figure S2 Assessment of the impact of including questionnaire data to maximize sample size versus potential misclassification. A total of 49 lipid-trait associated SNPs were tested for an association with gallstone disease using logistic regression adjusted for age, sex, and body mass index [kg/m^2^]. The analysis was performed twice where cases and controls were 1) defined by questionnaire *or* ultrasound data and 2) defined by questionnaire *and* ultrasound data. Synthesis-View [26] was used to display the results. SNP location (genome build 37.5) is given on the x-axis and p-values (-log_10_ transformed) are plotted along the y-axis for the top of the figure, while case/control totals are plotted along the y-axis for the bottom of the figure. Each triangle represents a p-value and each full circle represents a case while each empty circle represents a control. Triangles are color-coded such that red represents results from cases and controls defined by questionnaire *or* ultrasound data and blue represents results from cases and controls defined by questionnaire *and* ultrasound data. The direction of the arrows corresponds to the direction of the beta coefficient. The significance threshold is indicated by the red bar at p = 0.05. Click here for file
