# Supplementary material for: Lipid trait-associated genetic variation is associated with gallstone disease in the diverse Third National Health and Nutrition Examination Survey (NHANES III)
Source: BMC Med Genet. 2013 Nov 21;14:120. doi: 10.1186/1471-2350-14-120 (PMC3870971; doi:10.1186/1471-2350-14-120)
Supplement: Additional file 1: Table S1. — All tests of association for gallstone disease by population. Single SNP tests of association were performed using logistic regression assuming an additive genetic model. Results displayed here were adjusted for age, sex, and body mass index. SNP position is based on genome build 37.5. [file 1471-2350-14-120-S1.doc]

**Additional file 1: Table S1. All tests of association for gallstone disease by population.** Single SNP tests of association were performed using logistic regression assuming an additive genetic model. Results displayed here were adjusted for age, sex, and body mass index. SNP position is based on genome build 37.5.

| **SNPs** | **CHR** | **Position** | **Nearest Gene** | **Coding Function** | **OAT** | **Non-Hispanic Whites** | | **Non-Hispanic Blacks** | | **Mexican American** | |
| --- | --- | --- | --- | --- | --- | --- | --- | --- | --- | --- | --- |
|  |  |  |  |  |  | **OR (95% C.I.)** | **P** | **OR (95% C.I.)** | **P** | **OR (95% C.I.)** | **P** |
| rs11206510 | 1 | 55496039 | *PCSK9* | - | LDL | 1.07 (0.88,1.3) | 5.27E-01 | 1.19 (0.86,1.65) | 2.96E-01 | 1.09 (0.81,1.48) | 5.60E-01 |
| rs1748195 | 1 | 63049593 | *DOCK7* | intronic 3' down-stream | TG | 0.9 (0.77,1.05) | 1.80E-01 | 0.87 (0.68,1.1) | 2.42E-01 | 1.17 (0.95,1.45) | 1.41E-01 |
| rs10889353 | 1 | 63118196 | *DOCK7* | intronic | TG | 0.9 (0.76,1.05) | 1.83E-01 | 0.84 (0.67,1.06) | 1.50E-01 | 1.32 (1.05,1.67) | 1.73E-02 |
| rs12740374 | 1 | 109817590 | *CELSR2* | 3utr non-coding 3' down-stream | LDL | 1.03 (0.85,1.24) | 7.71E-01 | 1.14 (0.87,1.49) | 3.36E-01 | 1.14 (0.88,1.47) | 3.12E-01 |
| rs646776 | 1 | 109818530 | *CELSR2* | 3' down-stream | LDL | 1.06 (0.87,1.29) | 5.59E-01 | 1.08 (0.85,1.37) | 5.42E-01 | 1.1 (0.84,1.43) | 4.84E-01 |
| rs2144300 | 1 | 230294916 | *GALNT2* | intronic | HDL | 0.86 (0.73,1.01) | 6.06E-02 | 0.92 (0.69,1.24) | 5.94E-01 | 0.9 (0.73,1.1) | 2.90E-01 |
| rs7557067 | 2 | 21208211 | *APOB* | - | TG | 1.08 (0.89,1.31) | 4.26E-01 | 1.08 (0.78,1.48) | 6.55E-01 | 0.81 (0.63,1.02) | 7.40E-02 |
| rs515135 | 2 | 21286057 | *APOB* | - | LDL | 0.96 (0.79,1.16) | 6.67E-01 | 0.87 (0.69,1.09) | 2.10E-01 | 0.9 (0.69,1.17) | 4.33E-01 |
| rs562338 | 2 | 21288321 | *APOB* | - | LDL | 1.02 (0.84,1.24) | 8.40E-01 | 1.1 (0.88,1.38) | 4.18E-01 | 1.02 (0.77,1.36) | 8.91E-01 |
| rs754523 | 2 | 21311691 | *APOB* | - | LDL | 1.02 (0.86,1.2) | 8.34E-01 | 1.36 (1.04,1.78) | 2.41E-02 | 0.86 (0.68,1.08) | 1.82E-01 |
| rs780094 | 2 | 27741237 | *GCKR* | intronic | TG | 0.93 (0.79,1.09) | 3.55E-01 | 0.91 (0.67,1.24) | 5.53E-01 | 0.98 (0.79,1.22) | 8.73E-01 |
| rs6756629 | 2 | 44065090 | *ABCG5 ABCG8* | coding-nonsyn | LDL | 1.89 (1.44,2.49) | 1.00E-04 | 1.24 (0.82,1.87) | 3.03E-01 | 1.19 (0.85,1.67) | 3.04E-01 |
| rs6544713 | 2 | 44073881 | *ABCG8* | intronic | LDL | 1.35 (1.14,1.61) | 7.00E-04 | 1.19 (0.88,1.62) | 2.65E-01 | 1.22 (0.92,1.6) | 1.66E-01 |
| rs12695382 | 3 | 118948171 | *B4GALT4* | 3' down-stream non-coding intronic | LDL | 0.85 (0.68,1.06) | 1.53E-01 | 0.9 (0.56,1.43) | 6.43E-01 | 1.38 (0.91,2.1) | 1.27E-01 |
| rs1501908 | 5 | 156398169 | *TIMD4* | - | LDL | 0.98 (0.83,1.15) | 7.86E-01 | 0.82 (0.65,1.03) | 8.37E-02 | 0.89 (0.71,1.13) | 3.41E-01 |
| rs714052 | 7 | 72864869 | *BAZ1B* | intronic | TG | 0.97 (0.76,1.23) | 7.82E-01 | 1.09 (0.64,1.85) | 7.48E-01 | 1.05 (0.71,1.55) | 8.26E-01 |
| rs17145738 | 7 | 72982874 | *TBL2* | 3' down-stream | TG | 1 (0.78,1.27) | 9.77E-01 | 1.17 (0.77,1.8) | 4.63E-01 | 1.11 (0.75,1.65) | 5.95E-01 |
| rs2197089 | 8 | 19826373 | *LPL* | 3' down-stream | HDL TG | 1.04 (0.89,1.22) | 6.31E-01 | 1 (0.76,1.31) | 9.89E-01 | 0.98 (0.8,1.2) | 8.68E-01 |
| rs12678919 | 8 | 19844222 | *LPL* | - | HDL | 1.26 (0.95,1.67) | 1.14E-01 | 0.98 (0.69,1.39) | 9.08E-01 | 1.06 (0.66,1.7) | 8.04E-01 |
| rs10503669 | 8 | 19847690 | *LPL* | - | HDL TG | 0.81 (0.61,1.07) | 1.35E-01 | 1.44 (0.93,2.21) | 1.01E-01 | 0.83 (0.53,1.32) | 4.35E-01 |
| rs6586891 | 8 | 19914598 | *LPL* | - | HDL TG | 1.07 (0.91,1.25) | 4.06E-01 | 0.9 (0.67,1.2) | 4.72E-01 | 1.12 (0.91,1.36) | 2.89E-01 |
| rs2954029 | 8 | 126490972 | *RP11-136O12.2* | non-coding intronic | TG | 0.95 (0.82,1.1) | 4.97E-01 | 1.15 (0.9,1.47) | 2.68E-01 | 1.08 (0.88,1.32) | 4.73E-01 |
| rs471364 | 9 | 15289578 | *TTC39B* | non-coding intronic | HDL | 1.12 (0.87,1.45) | 3.79E-01 | 1.09 (0.81,1.47) | 5.79E-01 | 0.94 (0.65,1.37) | 7.40E-01 |
| rs4149268 | 9 | 107647220 | *ABCA1* | intronic | HDL | 1.1 (0.94,1.3) | 2.34E-01 | 1.03 (0.81,1.31) | 8.21E-01 | 0.75 (0.6,0.95) | 1.53E-02 |
| rs3905000 | 9 | 107657070 | *ABCA1* | intronic | HDL | 1 (0.8,1.26) | 1.00E+00 | 0.88 (0.64,1.21) | 4.16E-01 | 0.71 (0.47,1.06) | 8.99E-02 |
| rs1883025 | 9 | 107664301 | *ABCA1* | intronic | HDL | 1.09 (0.92,1.3) | 3.36E-01 | 0.84 (0.66,1.09) | 1.86E-01 | 0.78 (0.62,0.98) | 3.30E-02 |
| rs174547 | 11 | 61570783 | *FADS1* | non-coding intronic 3' down-stream | HDL | 1.1 (0.93,1.29) | 2.76E-01 | 1.24 (0.87,1.77) | 2.45E-01 | 1.07 (0.86,1.33) | 5.48E-01 |
| rs28927680 | 11 | 116619073 | *BUD13* | 3utr | HDL TG | 1.23 (0.93,1.62) | 1.53E-01 | 1.4 (1.06,1.86) | 1.92E-02 | 1.07 (0.81,1.42) | 6.27E-01 |
| rs964184 | 11 | 116648917 | *ZNF259* | 3utr | TG | 0.94 (0.76,1.16) | 5.55E-01 | 0.78 (0.6,1.01) | 6.37E-02 | 0.94 (0.76,1.17) | 6.05E-01 |
| rs12286037 | 11 | 116652207 | *ZNF259* | 3' down-stream intronic | TG | 0.84 (0.63,1.13) | 2.47E-01 | 0.77 (0.59,1.01) | 6.25E-02 | 1.01 (0.75,1.36) | 9.56E-01 |
| rs2338104 | 12 | 109895168 | *KCTD10* | 5utr non-coding intronic | HDL | 0.9 (0.77,1.04) | 1.53E-01 | 1.13 (0.88,1.45) | 3.53E-01 | 1.04 (0.85,1.28) | 6.82E-01 |
| rs2650000 | 12 | 121388962 | *HNF1A LEF1* | - | LDL | 1.15 (0.97,1.35) | 1.02E-01 | 1.5 (1.02,2.21) | 3.73E-02 | 0.98 (0.79,1.2) | 8.21E-01 |
| rs4775041 | 15 | 58674695 | *LIPC* | - | HDL TG | 0.99 (0.84,1.17) | 9.44E-01 | 1.22 (0.9,1.66) | 2.05E-01 | 1.25 (0.98,1.61) | 7.49E-02 |
| rs10468017 | 15 | 58678512 | *LIPC* | - | HDL | 1.01 (0.86,1.2) | 8.96E-01 | 0.87 (0.65,1.16) | 3.40E-01 | 0.88 (0.68,1.13) | 3.17E-01 |
| rs12596776 | 16 | 56919348 | *SLC12A3* | intronic | HDL | 0.83 (0.64,1.08) | 1.64E-01 | 0.89 (0.55,1.46) | 6.51E-01 | 1.06 (0.7,1.59) | 7.87E-01 |
| rs9989419 | 16 | 56985139 | *HERPUD1* | - | HDL | 0.93 (0.79,1.09) | 3.68E-01 | 0.88 (0.7,1.11) | 2.90E-01 | 1 (0.81,1.24) | 9.95E-01 |
| rs3764261 | 16 | 56993324 | *CETP* | - | HDL | 0.95 (0.8,1.12) | 5.15E-01 | 0.71 (0.56,0.9) | 4.70E-03 | 1.06 (0.85,1.33) | 5.89E-01 |
| rs1566439 | 16 | 57024662 | *NLRC5* | non-coding intronic | HDL | 0.99 (0.85,1.15) | 8.65E-01 | 0.86 (0.66,1.13) | 2.89E-01 | 1.06 (0.87,1.28) | 5.78E-01 |
| rs2271293 | 16 | 67902070 | *NUTF2* | intronic | HDL | 1.12 (0.89,1.4) | 3.42E-01 | 1.31 (0.87,1.99) | 2.02E-01 | 1.17 (0.89,1.54) | 2.63E-01 |
| rs4939883 | 18 | 47167214 | *LIPG* | - | HDL | 1.28 (1.03,1.59) | 2.71E-02 | 0.94 (0.75,1.19) | 6.17E-01 | 0.88 (0.65,1.19) | 4.14E-01 |
| rs2156552 | 18 | 47181668 | *LIPG* | - | HDL | 1.12 (0.89,1.4) | 3.25E-01 | 0.76 (0.46,1.26) | 2.91E-01 | 0.8 (0.53,1.19) | 2.65E-01 |
| rs2967605 | 19 | 8469738 | *RAB11B MARCH2* | 3' down-stream | HDL | 0.93 (0.76,1.14) | 4.77E-01 | 1.08 (0.82,1.41) | 6.06E-01 | 0.98 (0.78,1.23) | 8.49E-01 |
| rs10401969 | 19 | 19407718 | *SUGP1* | intronic | TG | 1.37 (1.05,1.78) | 1.89E-02 | 1.01 (0.74,1.38) | 9.61E-01 | 0.69 (0.41,1.15) | 1.52E-01 |
| rs16996148 | 19 | 19658472 | *CILP2* | 3' downstream | LDL TG | 0.81 (0.62,1.05) | 1.08E-01 | 1.1 (0.79,1.52) | 5.80E-01 | 1.12 (0.7,1.77) | 6.39E-01 |
| rs17216525 | 19 | 19662220 | *CILP2* | - | TG | 0.76 (0.58,0.99) | 4.45E-02 | 1.26 (0.64,2.46) | 5.02E-01 | 0.88 (0.53,1.45) | 6.15E-01 |
| rs4420638 | 19 | 45422946 | *APOC1* | 3' down-stream | LDL | 1.07 (0.87,1.31) | 5.46E-01 | 1.19 (0.89,1.59) | 2.44E-01 | 0.82 (0.58,1.15) | 2.43E-01 |
| rs6102059 | 20 | 39228784 | *MAFB* | - | LDL | 1.05 (0.88,1.24) | 5.99E-01 | 0.98 (0.78,1.22) | 8.51E-01 | 0.91 (0.72,1.15) | 4.29E-01 |
| rs1800961 | 20 | 43042364 | *HNF4A* | coding-nonsyn 3utr | HDL | 0.76 (0.48,1.2) | 2.34E-01 | 3.09 (0.41,23.28) | 2.74E-01 | 0.6 (0.36,1) | 5.17E-02 |
| rs7679 | 20 | 44576502 | *PCIF1* | intronic | HDL | 0.97 (0.79,1.19) | 7.45E-01 | 1.18 (0.75,1.84) | 4.73E-01 | 1.51 (1.13,2.01) | 4.80E-03 |

Abbreviations: chromosome (CHR); original associated trait (org. assoc.); odds ratio (OR); confidence interval (CI); p-value (P)
