# Supplementary material for: Lipid trait-associated genetic variation is associated with gallstone disease in the diverse Third National Health and Nutrition Examination Survey (NHANES III)
Source: BMC Med Genet. 2013 Nov 21;14:120. doi: 10.1186/1471-2350-14-120 (PMC3870971; doi:10.1186/1471-2350-14-120)

**Additional file 2: Figure S1. All associations between lipid trait-associated SNPs and gallstone disease by population.**


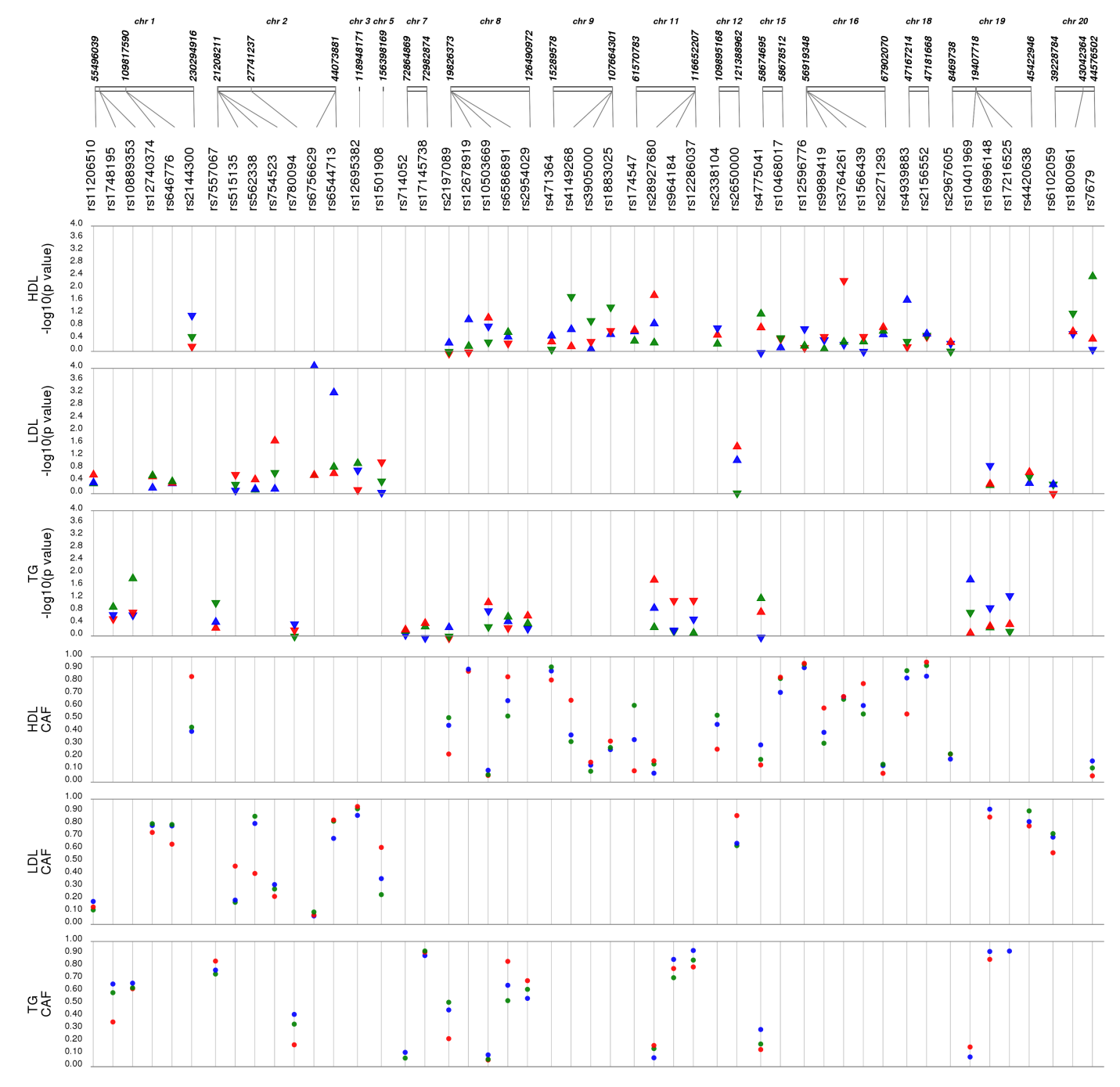

Supplement: Additional file 2: Figure S1 — All associations between lipid trait-associated SNPs and gallstone disease by population. A total of 49 lipid-trait associated SNPs were tested for an association with gallstone disease using logistic regression adjusted for age, sex, and body mass index [kg/m2]. Synthesis-View [26] was used to display the results. SNP location (genome build 37.5) is given on the x-axis and p-values (-log10 transformed) are plotted along the y-axis at the top of the figure, while coded allele frequencies (CAF) are plotted along the y-axis at the bottom of the figure. Each triangle represents a p-value and each circle represents a CAF for each race-ethnicity. Populations are color-coded as follows: non-Hispanic whites (blue), non-Hispanic blacks (red), and Mexican Americans (green). The direction of the arrows corresponds to the direction of the beta coefficient. The significance threshold is indicated by the red bar at p = 0.05. [file 1471-2350-14-120-S2.doc]
