# Supplementary material for: Lipid trait-associated genetic variation is associated with gallstone disease in the diverse Third National Health and Nutrition Examination Survey (NHANES III)
Source: BMC Med Genet. 2013 Nov 21;14:120. doi: 10.1186/1471-2350-14-120 (PMC3870971; doi:10.1186/1471-2350-14-120)
Supplement: Additional file 3: Figure S2 — Assessment of the impact of including questionnaire data to maximize sample size versus potential misclassification. A total of 49 lipid-trait associated SNPs were tested for an association with gallstone disease using logistic regression adjusted for age, sex, and body mass index [kg/m2]. The analysis was performed twice where cases and controls were 1) defined by questionnaire or ultrasound data and 2) defined by questionnaire and ultrasound data. Synthesis-View [26] was used to display the results. SNP location (genome build 37.5) is given on the x-axis and p-values (-log10 transformed) are plotted along the y-axis for the top of the figure, while case/control totals are plotted along the y-axis for the bottom of the figure. Each triangle represents a p-value and each full circle represents a case while each empty circle represents a control. Triangles are color-coded such that red represents results from cases and controls defined by questionnaire or ultrasound data and blue represents results from cases and controls defined by questionnaire and ultrasound data. The direction of the arrows corresponds to the direction of the beta coefficient. The significance threshold is indicated by the red bar at p = 0.05. [file 1471-2350-14-120-S3.doc]

**Additional file 3: Figure S2. Assessment of the impact of including questionnaire data to maximize sample size versus potential misclassification.**

**
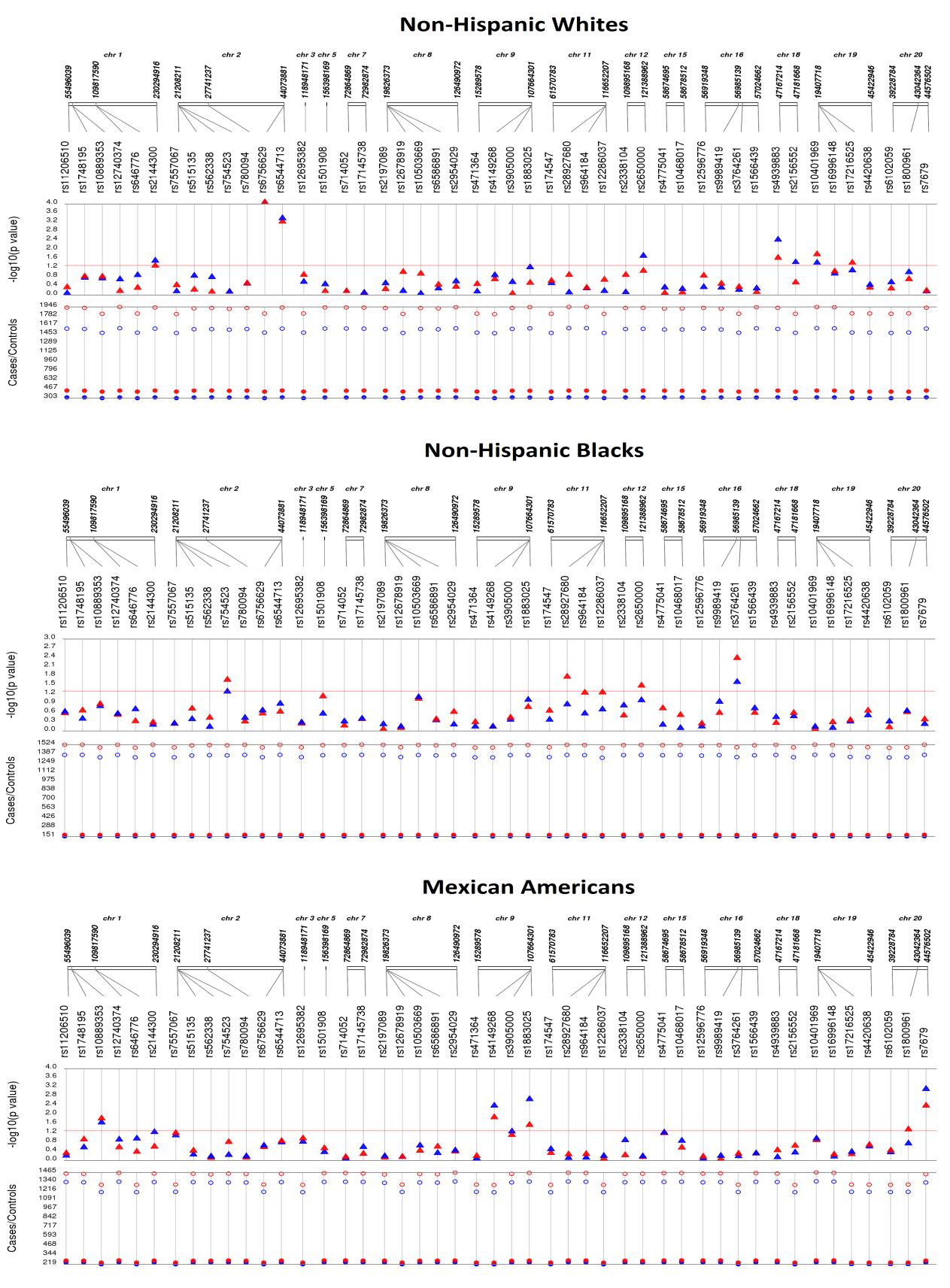
**
